# Time in refugee studies: a conceptual literature review and prospects from a social theory lens

**DOI:** 10.1186/s41018-026-00196-1

**Published:** 2026-06-06

**Authors:** Nanor Karageozian

**Affiliations:** Independent academic researcher, Montreal, Canada

**Keywords:** Refugees, Migration, Migration and refugee studies, Time, Temporality, Temporal dimensions, Social theory

## Abstract

Within social sciences, the concept of time has gained some attention in migration studies, among other fields, especially in the last couple of decades. While a few articles and books have focused on reviewing and conceptualizing the time dimension within the broader field of migration studies, they have rarely focused specifically on the subfield of refugee studies, even though many works in this subfield, mostly of empirical nature, have touched upon the concept of time explicitly or implicitly. This paper first reflects on what unpacking the time dimension would add to the study of refugee experiences and policies. Subsequently, the paper examines how some key conceptualizations of time in social theory have been addressed in and are relevant for some selected works within refugee studies. It specifically focuses on reviewing how different dimensions of time – timing; sequence (or order); frequency (or rhythm), including (a)synchronization; speed (or tempo); duration; and past, present and future – manifest at the micro, meso and macro levels of the social world pertaining to forced displacement. Recognizing the intertwined nature of these levels, the paper ends with some brief initial reflections on why and how to advocate for a balanced approach between mobility and immobility, agency and structure, informality and formality, and haphazardness and purposiveness, when studying or trying to understand temporalities in refugee lives and policies.

## Introduction

Within social sciences, the concept of time has gained some attention in migration studies, among other fields, especially in the last couple of decades. While a few studies have focused on reviewing and conceptualizing the temporal dimension within the broader field of migration studies (e.g. Griffiths et al. 2013; King et al. 2004; Tefera and Gamlen 2024), no similar conceptual reviews have specifically zoomed into the subfield of refugee studies, even though many works in this subfield, mostly of empirical nature, have touched upon the concept of time explicitly or implicitly. Thus, a dedicated, conceptually oriented review of temporality for refugee studies remains limited.

This paper first reflects on what unpacking the time dimension would add to the study of refugee experiences and policies. It explains why the subfield of refugee studies is important to analyse temporal dimensions, mainly related to the forced-migration-specific power relations (e.g. status precarity, legal time, deportability, elaborate humanitarian system). Subsequently, inspired by the above-mentioned review works in migration studies – particularly that of Griffiths et al. (2013) – the paper examines how some key conceptualizations of time in literary and especially social theory have been addressed in and are relevant for some works within refugee studies. Thus, the paper adopts a refugee studies lens to previously conducted reviews. It does so by reviewing how core dimensions of time – timing; sequence (or order); frequency (or rhythm), including (a)synchronization; speed (or tempo); duration; and past, present and future – manifest at the micro, meso and macro levels of the social world pertaining to forced displacement. Effort is also made to identify or highlight how these different aspects of temporality – which are often examined separately – are interconnected.

While the paper does not claim or aim to provide an exhaustive literature review or comprehensive analysis of the full spectrum of all relevant available literature, it is hoped that it will offer a stocktaking of some main selected works that explicitly tackle the core temporal dimensions, with the goal of demonstrating the patterns in which these dimensions are manifested in academic scholarship, while also advancing further conceptualization attempts through a temporal lens. The last section aims to provide some directions in this regard. Recognizing the intertwined nature of individual refugee biographies (micro), meso relations, and macro regimes/systems, the section presents brief initial reflections on why and how to advocate for a balanced approach between mobility and immobility, agency and structure, informality and formality, and haphazardness and purposiveness, when studying temporalities in refugee lives and policies. It thus hopes to advance a framework attentive to both structural constraints and refugees’ temporal agency.

## Concept of time in the broader field of migration studies

A few articles and books have been particularly important in reviewing and conceptualizing the concept of time in migration studies.

Two of the early efforts were those of Swedish geographer Torsten Hägerstrand^1^ and of Brazilian sociologist Saulo Cwerner.^2^ Their key contributions are summarized in King et al. (2004), who provided a detailed review of how the concept of time has been addressed in migration and integration studies. King et al. (2004, p. 15) also attempted to adopt “a more pragmatic stance” by “setting out a range of methodologies for studying migration and migrants from a temporal perspective.” They particularly highlighted the importance of the life-course approach and longitudinal studies. They also studied other cross-cutting issues, such as gender, age and family.

The next main relevant work came almost a decade later by Griffiths et al. (2013). They conducted a scoping study of what migration can add to studies of time (especially time in social theory) and, vice versa, how time has been dealt with explicitly and implicitly in studies of migration, particularly focusing on two areas of study: mobilities literature and life-course and longitudinal studies.

A few years after this important review piece, in the introductory chapter of their 2017 edited book *Timespace and International Migration*, Page et al. (2017) made reference to some recently published research works on the topic of time in migration studies. Despite these developments, they claimed that “the idea that time is under-analysed in migration studies remains salient”; however, they added that “temporality is emerging as a key theme in current thinking about migration” (ibid., pp. 3–4). In their edited book, they aimed to apply the conceptual discussions around the term “timespace” – particularly “May and Thrift’s four ‘domains’ of timespace (the rhythms of the global environment, the temporal effects of systems of social discipline, time-related technologies and, finally, texts about time)” (ibid., p. 4) – to the field of migration studies. However, they also recognized that time and space might really be different, and thus studying them separately after having “internalized the idea of timespace” is also important (ibid., pp. 6–8).

More recently, Tefera and Gamlen (2024) reviewed some works that have applied a temporal lens to migration and refugee research within human geography and related disciplines, such as sociology and anthropology. They outlined three approaches in the recent literature: the “displacement and mobilities approach,” the “transit and waiting approach,” and the “immigrant settlement approach.” They argued that these approaches “often reify problematic temporal categories of ‘past,’ ‘present,’ and ‘future,’ leading to linear and Western-centric views of migrant experiences” (Tefera and Gamlen 2024, p. 863).

## Concept of time in refugee studies: Why is it relevant and important?

Within the field of refugee studies specifically, time surfaces explicitly and implicitly as a key concept or aspect in several studies. Yet, to the best of my knowledge, no dedicated or targeted review studies have been undertaken focusing specifically on refugee studies, despite the above-mentioned efforts to review the temporal dimension within the broader field of migration studies. In most of the above-mentioned works, limited reference is made to refugee studies in specific. Griffiths et al. (2013) did provide some examples related to asylum seekers and refugees in different parts of their review study. Also, King et al. (2004, pp. 62–64) referred to refugee and asylum-seeking children. A couple of chapters in the book *Timespace and International Migration* – such as those by Griffiths (2017) and by Novak (2017) – are also relevant to refugee studies. Among the above-mentioned works, Tefera and Gamlen (2024)’s paper, which builds partly on their previous research on refugee-background Ethiopians in Melbourne, Australia (Tefera and Gamlen 2023), touches upon forced displacement-related issues and examples the most.

This paper aims to fill a part of this gap, by focusing on the growing relevant literature specifically in refugee studies. But before doing so, it is worth reflecting on what unpacking the time dimension would add to the study of refugee experiences and policies. To answer this question, I build upon some of the lines of thought included in Mozetič’s (2018, pp. 235–236) work, where she made linkages with social identity theory in her study about the self-perceptions of non-European refugee physicians in Sweden.

In his theory of social identity, Jenkins (2000, pp. 10–13) defined society or the social world “as a set of relationships within and between three ‘orders’ of social phenomena”:

1. The “individual order” (individual human beings and their beliefs, perceptions, “view[s] of selfhood,” etc.);
2. The “interaction order” (how different individuals co-exist and their relationships, their practices of interacting with each other); and
3. The “institutional order” (that of institutions or organizations, “the world of patterned, organized and symbolically-templated ‘ways-of-doing-things’” that is “embodied in buildings, territories, visible symbolism.” According to this order, “decisions are made, action oriented, and resources and penalties distributed”).

As Jenkins (2000) recognized, these three orders are highly linked to and overlap with one another. From a refugee studies lens, the boundaries are blurred between the micro level or scope of events/influence in an individual refugee’s personal life, the meso level of his/her relationships with others (e.g. family, groups, networks), and the macro level of refugee policies and practices by humanitarian organizations, donors, political parties, and states at large. It is in the complex processes through which these different levels relate to one another over time, i.e. as “individuals [are] interacting in institutionalized contexts” (ibid., p. 14), that (social) identity is made and re-made, produced and reproduced. More specifically, Jenkins (2000, pp. 9, 11) recognized the importance of viewing “self- and group identification” (which he conceived as an internally oriented process) and “social categorization” (considered as an externally oriented process) – or “self-image” and “public image” – as dynamic processes that evolve over time. Thus, by understanding how time is perceived and is manifested in the three orders or levels, as well as how these perceptions or manifestations change over time can give us a better understanding of (social) identity and its evolution. Put differently, a temporal dimension allows us to unpack the relationship between the different levels – their mismatches and overlaps – in an effort to better understand identifications and categorizations, and their evolutions.

This, of course, is not only relevant to refugee studies. As Griffiths et al. (2013, p. 30) argued in their review of time in migration studies in general:

Individual subjectivities and nation states do not map seamlessly on to [sic.] each other. Of particular interest is the conflict between the institutional or bureaucratic time of the state, which ‘claims to be absolute, universal, total’ and individual time ‘which is personal, quotidian, limited’ … This tension pervades our multiple understandings of time, and migration, understood as mobility and residence to/in a territory of which one is not a citizen, brings it very much to the fore.

However, the field of refugee studies presents an important sub-field to analyse temporal dimensions from the above-explained perspective, because of its special characteristics relative to other forms of international migration, including the forcible and often sudden triggers of refugee flows from their places of residence, the difficulty and often impossibility to return to these places (at least for certain and sometimes for extended periods of time), the quite well-established and expanded system of humanitarian organizations dealing with refugee issues, the often highly politicized and regulated nature of refugee flows and (re)settlements in the international refugee regime, etc.^3^ As Cangià et al. (2021, p. 58) mentioned, referring to some of these characteristics:

The migration of refugees represents an interesting context for exploring time disruption and the interplay between social and personal time: refugees are forcibly displaced and do not always have the time to prepare for departure, and face numerous bureaucratic constraints relating to the temporariness and length of their stay in the country.

Thus, a temporal lens in refugee studies is particularly helpful in illuminating the intersection of individual refugee experiences and identities, their social relations, and institutional settings, as explained below.

## Temporal variables for explaining relations between micro, meso and macro levels in refugee studies

This section explains how some key conceptualizations of time in literary and especially social theory have been addressed in and are relevant for some works within refugee studies. These temporal variables can help explain the relationships between the micro, meso and macro levels of the social world pertinent to refugee studies.

In her work on the “timescapes perspective,” sociologist Barbara Adam (1998, 2004) aimed to examine the complexities and various dimensions of time. She specifically identified the following elements of a timescape: “time frame”, “temporality”, “timing”, “tempo”, “duration”, “sequence” and “temporal modalities (past, present, future)” (Adam 2008).^4^  

In the field of literary theory, Gérard Genette’s work on narratology focused on three concepts of time to understand the relationship between narration and story: order, duration and frequency (Guillemette and Lévesque, n.d.; Martin 2016).

How have such temporal dimensions informed time-oriented analyses in different fields of studies relevant to refugee studies?

Exploring time in migration studies, Griffiths et al. (2013, pp. 14–29) focused on five main temporal categories: “flows and moments; rhythms and cycles; tempos; synchronicity and disjuncture; and the future.” They reviewed how each category has been addressed in migration or related studies and proposed potential areas of further research.

Somehow similarly, Howlett and Goetz (2014, p. 486) looked at different dimensions of political time when examining the concept of time in the study of politics, administration and policy. They argued that:

Political time is institutionalised, in particular, through rules that govern the length and configuration of terms, mandates and tenures of elected and un-elected officials; through rules that determine when (timing), in what order (sequence), how quickly (speed) and for how long (duration) actions can be taken; and at the level of policy, which is concerned with allocation *in time* and *over time*. Formal time rules are complemented by more or less deeply entrenched informal rules…

In a similar vein, in most of the rest of this paper, I briefly explain how some core temporal variables are applicable to refugee studies specifically, citing some works that have addressed them. In doing so, I will also address different ways in which the micro, meso and macro levels interact. As noted above, the referenced works are just some selected examples; the aim is not to provide a comprehensive literature review but rather identify and demonstrate the different ways or recurring patterns through which core temporal dimensions are manifested or treated/analysed in academic scholarship. The selection of the reviewed works has been made by giving preference to those that: (1) treat time as a central or explicit focus of their analysis; and (2) include either an in-depth analysis of one of the core temporal variables examined or touch upon more than one temporal variable. In addition, the selection took into consideration geographical diversity of the empirical cases examined in the works, while also ensuring the inclusion of studies that focus on micro, meso and macro levels. The latter is particularly important as a conceptual point of departure for the reflections I include in Section An initial attempt to advocate for a balanced approach when conceptualizing temporalities in refugee studies: early exploratory reflections. By presenting scholarship to illuminate how time is reflected in or affects refugees’ individual lives and experiences, relations, and broader structural regimes and institutional systems – in mutually reinforcing and constitutive manners – allows me to start engaging in further conceptual debates about challenging and moving beyond binaries (mobility/immobility, agency/structure, informality/formality, and haphazardness/purposiveness) and in favour of or towards co-existing temporal logics.

My literature review has revealed that certain themes, such as “waiting”, have been addressed in a relatively larger number of studies compared to other temporal issues. This is to a certain extent reflected in my analysis below, where the sections dedicated to some of the temporal dimensions are longer or more elaborate than others.^5^ This in itself constitutes a useful contribution of the paper, as it can point to certain gaps and future research opportunities in scholarship.

While some variations exist in the typologies and especially the labeling of dimensions in different works, the variables that I examine in this paper cover all the core temporal dimensions identified by various authors, especially those common in migration temporal analyses; it builds on these syntheses but regrounds them in refugee studies. The temporal variables analysed in this paper were chosen because: (a) they recur across foundational social sciences-oriented studies of time (e.g. Adam 2008) and migration-related reviews (e.g. Griffiths et al. 2013), (b) they map onto policy-relevant processes, and (c) they enable bridging micro–meso–macro levels. I have adopted labels used by others or slightly adapted them, by taking into consideration what would make them most relevant or intuitively understood in relation to refugee studies.

On a related note, it is important to highlight that the temporal variables are analytically distinct but empirically intertwined. Thus, while timing, sequence, speed, etc. are examined under separate sections below, this is done for analytical purposes only; in lived experience, these temporalities are entangled. For instance, “waiting” entwines speed (deceleration), duration (indeterminacy), rhythm (disruption of routines), and future orientation (hope/anticipation). Even though the different variables have dedicated subsections, throughout the analysis, effort is made to identify or highlight how the different aspects of temporality – which are often examined separately in academic literature – are interconnected. The same applies to the distinction between micro, meso and macro levels. Micro biographies, meso relations, and macro systems or regimes are highly intertwined in reality (Jenkins 2000) – hence also this paper’s emphasis on helping surface, or explicitly highlight, their interconnections. In different parts of the analysis, I therefore point to some of these overlaps or connections, returning to their synthesis in my reflections in Section An initial attempt to advocate for a balanced approach when conceptualizing temporalities in refugee studies: early exploratory reflections.

### Timing

This variable is related to what Griffiths et al. (2013) identified as “moments”. It would include, for example, the way refugees describe and interpret the timing of their personal/family decision to flee their areas of origin/residence or that of other key events/decisions in their individual/family lives. The works of Schon (2019) and of Tarkhanova and Pyrogova (2024), for instance, touched upon the timing of the decisions of Syrians and Ukrainians to flee their homes during conflict, respectively.

From a macro perspective, this variable is related to how humanitarian organizations or governments define the start and other key events of a “refugee crisis”. In general, the timing of decisions or policies by such institutions is another example. The timing of institutional decisions is sometimes based on snapshot studies or assessments that collect data about refugees at specific points in time. Brun (2016b), for instance, explained the case of the Vulnerability Assessment Framework (VAF) that gathers information about Syrian refugees in Jordan to inform how assistance will be distributed.^6^ Brun (2016b, p. 401) argued that VAF “provides a generalized picture that only depicts the now. It is currently only helpful to assist individuals and households based on need in one particular moment and without understanding spatial or temporal contexts.” I will come back to Brun’s work later in this review, in the sub-section covering the temporal dimension of future.

### Sequence (or order) and frequency (or rhythm), including (a)synchronization

#### Sequence (or order)

In academic studies or analyses, often refugees’ lives and experiences are divided into phases that are sequenced in a specific way. For example, Mzayek (2019) divided her analysis into “four distinct temporal spaces in which narratives of wellbeing were experienced by the Syrian refugees”: “before the war,” “war and displacement”, “initial resettlement in intermediate countries” and “resettlement in Austin, Texas,” United States of America (USA). Khosravi (2020, p. 203) referred to “a temporal and spatial stretching of waiting from pre-departure, and transit, to post-arrival and even post-deportation”. Cangià et al. (2021, p. 60, emphasis in original) focused on waiting after relocation and divided it into: “1. *waiting for the asylum decision* (in the center and outside), 2. *waiting for the ‘right’ job* and 3. *waiting for the past to come back or the future to happen*”.

Although such sequencing is understandable, as it is often done for practical reasons to facilitate analysis, it might obscure complexities.

Firstly, these experiences are often narrated after some time has passed – that is, ex post facto – and thus refugees might perceive and describe certain phases often in relation or comparison to others retroactively, and not how they were felt or perceived at the time of occurrence.

In addition, in such sequential analyses, there is sometimes a degree of perceived finality or eventual end of phases, which are perceived in a unidirectional manner. Refugee resettlement is often regarded as the end of that line; thus, issues related to the post-resettlement might not receive enough attention. This is what Omar (2022) attempted to address in her study of Syrian refugee mothers who arrived in Canada. By adopting a temporal lens, she aimed to study the challenges that refugee mothers face after their resettlement, especially related to their conceptualization of their children’s and their own futures. She did so by introducing the concepts of “foreclosed futures” and “entangled timelines”.^7^

Also, (back-and-forth) movements, both physically and emotionally, between phases are often not covered comprehensively. For example, issues arising when refugee settlement and/or resettlement happen(s) in more than one destination over time, or when a refugee (extended) family is divided by settling/resettling into more than one destination, are often not addressed adequately. Furthermore, many refugees move multiple times – within countries, across regions, and between statuses – creating braided timelines that defy linear phase models. This seriality amplifies asynchronies (documents expiring out of phase with opportunities) and reconfigures rhythms (schooling/work cycles repeatedly interrupted), thus touching upon the “rhythm” dimension further examined below. It also shapes identity through iterative (re-)categorizations (registered/unregistered; temporary/provisional).

Moreover, variations within phases are often underemphasized. For instance, Mzayek (2019) identified the first phase as a phase of happiness, the second as suffering, the third as liminality characterized by identity loss/issues and uncertainty, and the fourth as initially a mixture of anxiousness with joy and relief, followed by challenges, mostly financial, linked to feelings of depression, anxiety and stress. Somehow similarly, Mozetič (2018, p. 240) explained that refugee doctors had a sense of insecurity in their initial period in Sweden, because of the uncertainties characterizing their lives and the feeling of dependence and powerlessness related to external circumstances. After receiving their resident permit and Swedish medical license, these feelings subsided, he argued.

The temporal dimension of sequence is also linked to how life-course cycles are perceived. While this topic is further discussed and elaborated upon under other dimensions, such as “frequency (rhythm)” and “duration”, it might be worth highlighting here the life-course disruption aspect of time in refugee studies works. For example, Grabska’s (2020) work explores how Eritrean adolescent girls and young women living as refugees in Khartoum, Sudan, navigate the gap between their aspirations of reaching a “better place” elsewhere and the reality of prolonged waiting and uncertainty in a transitory context. The girls’ narratives reveal how “dreams of being elsewhere, impossibilities of returning, and realities of uncertainties and being-stuck inbetween [sic.]” shape their understandings of becoming adults (ibid., p. 22). This demonstrates how adolescence in this refugee context is not a linear developmental stage, but a complex interplay of hope, frustration, agency and constraint, where gender, space and time intersect to influence how young refugee women make sense of their lives and futures.

From a more macro perspective, how events are ordered or are presented to be ordered affects “the public constitution of a ‘problem’,” “its identification with particular social categories – and their identification with it – and eventually ... the framing of policy responses” (Jenkins 2000, p. 19). Giving the example of social policy, Jenkins (ibid.) argued:

The targeting of resources and interventions at a section of the population which is perceived to have particularly urgent or specialized ‘needs’, may call into existence a new social categorization, or strengthen existing categorizations. This classification may emphasize the entitlement of those in question to receive resources. Equally, however, it may identify them as socially deficient, or lacking in some fashion, labelling them further as ‘undeserving’ or ‘troublesome’.

This also applies to refugee-related policies, with impacts on identity issues. For example, Mzayek (2019, pp. 374–375) explained how the provision of medical, housing and financial assistance during the first few months after resettlement in Austin made many refugees more hopeful about their futures, but once this initial period passed and assistance got reduced, they encountered multiple challenges that also affected their well-being and identity.

Assistance before and after refugee registration is another issue, where registration is viewed as a cutting point in time to determine access to certain types of refugee assistance, because it is only then that individuals are officially categorized as “refugees”, allowing international organizations to disperse resources and provide services.

#### Frequency (or rhythm)

Highly linked to the above-described temporal dimension of sequence (or order) is that of frequency or rhythm, which has to do with “regularly recurring or alternating phenomena” that highlight “the importance of repetition, simultaneity, seasonality and cycles” (Griffiths et al. 2013, p. 17). This concept is tackled, for example, by Novak (2017) in his book chapter on assistance for Afghan refugees over the border in Pakistan spanning three decades. Relying on the idea of “rhythmanalysis”, Novak’s study draws “attention to the changing rate of labour and refugee flows, the differentiated temporal experience of boundary-crossing and the coming-into-being of boundary lines themselves”, highlighting “the multiplicity of different tempos that can be heard simultaneously – beats that are distinct but related and which, together, produce the vast unity that we seek to disentangle”, as the book editors summarized (Page et al. 2017, p. 10).

At a micro (and meso) level, one issue related to this dimension is the impact that forced displacement might have on refugees’ life course and in general how it might interrupt or disrupt their regular life rhythm(s) or routines. In their study of young people growing up as refugees in Uganda and Jordan, coming from five different national groups and located in camp and urban settings, van Blerk et al. (2021) conceptualized “youth transitions to adulthood” in protracted crisis situations. They argued that such phenomena not only lead to delays, modifications or disruptions of certain life events in these people’s lives, but can lead to a “rupture” of their capabilities and future aspirations (ibid., p. 1). They defined “rupture” as “a severing of expected life pathways and an extirpation of past social and cultural connections, current opportunities, and future aspirations” (ibid., p. 2). They identified four main processes that affect the transitions of young refugees related to these rupturing situations: “the lasting traumatic experience of displacement; the temporal effects of lives lived on a ‘temporary’ basis, beyond waithood …; limits to the formation of social relations; and the institutional conditions that govern everyday life” (ibid., p. 7). Focusing on the experience of exile for Iraqi refugees in Cairo as “living in transit”, El-Shaarawi (2015, p. 45) described how this situation “constrained and disrupted Iraqis’ expected and imagined life trajectories” and “sometimes rendered important life milestones uncertain or impossible”. She gave the examples of disrupted education due to inability to afford the school or university fees, delayed marriages due to difficulty finding a suitable partner, and postponed childbirth due to the lack of financial resources or a family support system.

More explicitly linking micro and macro levels, Heidbrink (2022) argued that deportation operates not only as a form of legal, physical or structural violence but as a distinct form of “temporal violence”, through threat of sudden acceleration (arrest, removal) and extended uncertainty (appeals, monitoring) – violence that persists well beyond the moment of deportation, shaping everyday decision-making, family relations, and self-perception over time. Drawing on longitudinal, multisited ethnographic research with Indigenous Maya youth deported from the USA and Mexico to Guatemala, Heidbrink examined how deportation actively “forecloses futures”, compresses life into an unstable present, and disrupts long-term life projects, thereby forcing deported individuals – especially youth – to live in a perpetual “here and now.”^8^

One specific aspect of rhythm entails professional advancement. For example, there is often an acknowledgment of “how displacement disrupts or reshapes migrants’ conceptions of personal career time, from being linear, cumulative or simply stable to being uncertain and fragmented” (Cangià et al. 2021, p. 63). This is also linked to the dimension of duration, discussed below, because the sense of uncertainty often emanates from the expected or perceived permanence or temporariness of certain events or stages in refugees’ lives. For example, their feelings of uncertainty about their life course and how long its rhythm will stay disrupted may lead refugees to engage in precarious or unstructured or irregular work.

#### (A)synchronization

The temporal dimensions of sequence (or order) and frequency (or rhythm) are also related to the concepts of temporal “synchronicity” (or “simultaneity”) and temporal “asynchrony” (or “disjuncture” or “dissonance”); the latter can take the extreme form of “ruptures” (also mentioned above) or “tears” (Griffiths et al. 2013, pp. 23–26). As Griffiths et al. (2013, p. 23) explained in relation to migration studies in general, one aspect of synchronicity is that “a shared sense of time builds bonds between people and communities, establishing a sense of connection and preservation”. In relation to refugee studies, more specifically, the nostalgic feelings refugees often have about times/their lives before their displacement; the way they remember the time before their displacement, the time of their displacement, and the circumstances that happened at that time; or the way they recall their refugee journeys may form key elements of their refugee stories that might not only affect their individual identity but also contribute to the development of a common sense of belonging to a group. Focusing on the concept of “refugee journeys,” for example, Benezer and Zetter (2014, p. 303) argued:

On the individual level, the journey seems to effect the narrowing or expanding of personal boundaries, depending on its nature and the way it interacts with the individual’s culture and personality. On the group level, these journeys may have an effect on the way members of a migrating/fleeing society perceive themselves as a group, including their social identity, and on the ensuing expectations regarding the receiving society and its reception of them.

From a macro level, another aspect of temporal synchronicity or asynchrony is developing different policy responses towards refugees depending on the time they were displaced or settled in the host country, or alternatively, adopting uniform policy responses regardless of the special circumstances that might be potentially surrounding certain refugee waves or the uniqueness of individual refugee experiences, for example. In addition, the degree of simultaneity of policies and practices towards refugees by different humanitarian organizations might affect the level of their cooperation, reinforcing some potential synergies and trade-offs. These are in turn also linked to the micro and meso levels, that is, how these policies, practices and collaborative efforts might be juxtaposed to or might affect refugees’ individual identities and group identification (Fozdar and Hartley 2014; Hovil 2016; Zetter 1991), including across generations (Beirens et al. 2007; Fantino and Colak 2001), leading to social cohesion or tensions and conflicts.

The extreme asynchrony case of temporal rupture, which is “where the temporal normalcy of behaviour and the synchronization of social activities are usually *suddenly* interrupted,” as Cwerner (2001, p. 16) explained, is particularly relevant to refugee studies and “will not be typically part of the experience of other forms of migration.” This happens when, for example, people are suddenly forcibly displaced due to conflict, political instability, violence, disasters, etc.; or, when refugees receive their resettlement decision and need to resettle to a third country within a short period of time. In such circumstances, there is often an unexpected or quick mismatch – or “hysteresis” – between a refugee’s “habitus” and “field”, and hence a “time lag” (called “hysteresis effect”) is required for the habitus to reposition itself in the altered field, to use Pierre Bourdieu’s social theory concepts.^9^  

### Speed (or tempo)

Somehow related to the sudden nature of temporal ruptures described above is the temporal dimension of tempo, that is, the speed or velocity by which certain events occur in refugees’ lives. On the one hand, time might be considered as too fast – or what Griffiths et al. (2013, p. 19) called “acceleration” or “frenzied time”. On the other hand, it might be regarded as too slow, suspended or stagnant – or what these scholars dubbed “deceleration and stasis” (ibid., 2013, p. 20). For instance, “the pace of change, or speed of bureaucratic or legal processes are often considered problematic by those subject to them” (ibid., p. 18). Griffiths et al. (ibid.) gave the example of asylum decisions (with a focus on the British asylum system), which might be regarded as “both too slow and too fast.” The asylum system is often criticized for the red tape of its bureaucracy, leaving people waiting for long periods of time (Kobelinsky 2021). However, deportation decisions are sometimes made/announced too quickly or suddenly, leaving little time for people to plan or react.

From a macro perspective, “government rhetoric is often geared towards an assumption that speed is a sign of success …, with [m]inisters calling for deportations to occur at ever-quicker rates” (Griffiths et al. 2013, p. 20). Also from a macro perspective, this coexistence of acceleration and deceleration resonates with Andersson’s (2014) notion of a “temporal economics of illegality,” which shows how European migration control operates through postponement, anticipation, and deferred enforcement, rendering migrants perpetually deportable while economically exploitable. Time becomes a central resource for managing deportability, exploitation, and hierarchical inclusion. A related topic is the different pace at which certain refugee-related policies are adopted/enacted, often in distinct ways for different refugee groups/in different periods. Such debates have recently arisen, for example, in relation to the European Union (EU)’s 2022 activation of the Temporary Protection Directive (TPD) for people fleeing Ukraine, which created an immediate, time-bound protection regime with residence, work and schooling rights. While laudable by many for speed and scale, some scholarship and advocacy debates have highlighted its exceptionalism and racialized selectivity when contrasted with slower, more restrictive responses to other displaced populations. Thus, analytically, the TPD has been regarded by some as an example of how states can rapidly re-time protection through emergency instruments, revealing the politics of speed, duration and categorization (e.g. Corcodel et al. 2023; Ineli-Ciger 2022; Näre and Tkach 2024).

In terms of slow time, several studies in migration studies in general and refugee studies in specific address migrants’ or refugees’ sense of “suspension of time whilst the world around them continues forward” (Griffiths et al. 2013, p. 20). This relates to ideas of stillness and passivity in works that highlight how refugees wait for certain changes or decisions, how they queue for certain services for long periods of time, how they face slow bureaucracies (related to asylum-seeking, for example, as explained above), etc. The effects that these experiences of slow time have on feelings of liminality and idleness, and hence general well-being or suffering, are often highlighted. For example, El-Shaarawi (2015, p. 51) described the case of Iraqi refugees in Syria while they wait for their resettlement decision that could happen any time, but often took longer than expected. Brun (2015) focused on the case of Abkhazian internally displaced persons (IDPs) and their queuing for water or toilet in collective shelters, or to receive assistance from or solve other issues with the help of the responsible ministry. She described how they expressed being bored and watched TV to spend time, with boredom being linked to the slowing down or stillness of time.

Linking more the micro to the macro levels and based on longitudinal ethnographic research with 26 asylum seekers from seven countries in the USA, Haas (2017) argued that the United States (U.S.) asylum system produces applicants as simultaneously “citizens-in-waiting” and “deportees-in-waiting”, a dual and unstable positionality that is fundamentally temporal. Through prolonged, uncertain waiting, asylum seekers inhabit what she calls an “existential limbo” – a condition in which life is suspended, futures are inaccessible, and suffering becomes temporally organized by the state rather than confined to past persecution or future deportation. The article shows that waiting itself is a technique of power: the asylum process governs people not only through decisions, but through time – delay, uncertainty and indeterminacy – reshaping subjectivity, agency, and meaning-making.

### Duration

As mentioned above, the temporal dimension of speed, particularly related to slow time, is highly linked to the dimension of duration,^10^ that is, how long this slow time will or is expected to last and in general how permanent or temporary certain refugee-related circumstances or events will or are expected to be – both at micro and macro levels.

Here, again the concept of “waiting” comes to the fore and many works in the area of refugee studies address this issue explicitly.^11^ Mzayek (2019, p. 369) defined waiting as the time “being between flight and resettlement”. Focusing her research on a small community of Syrian refugees resettled in Texas, USA, Mzayek (ibid.) conceptualized it as a “transitory period of liminality, encompassing ambivalence in time and space, as well as a state of in-betweenness”. She added: “Within a liminal existence, refugees or migrants experience living in a space of uncertainty or in limbo” (ibid., p. 370).^12^ Horst and Grabska (2015) also referred to liminality in relation to protracted displacement. Brun (2015, p. 22) summarized Horst’s and Grabska’s focus on liminality as follows: It “captures the simultaneous process of marginalization, control, and stasis, on the one hand, and the transformation and flows, on the other.”

Prolonged waiting is often associated with a sense of “long-term uncertainty” (El-Shaarawi 2015) or “protracted uncertainty” (Horst and Grabska 2015). This is the case, for example, of refugees who find themselves residing for increasingly long periods of time in camp and urban settings in the Global South, often near their country of origin, while they wait for their resettlement, due to the “shrinking global asylum space and global inequities in responsibility sharing around refugees and other placed populations” (El-Shaarawi 2015, pp. 38–39). Such refugees often find themselves “stuck” for years in these countries that were supposed to be just transition points in their journey towards resettlement; they have no ability to return to their countries of origin, no interest in or possibility of meaningfully integrating in these host countries, and no option to move and settle elsewhere. Focusing on Iraqi refugees in Egypt, El-Shaarawi (2015, p. 39) applied the concept of uncertainty to examine “how refugees may experience exile as a kind of in-between, transit state, even when displacement spans a period of years.” This has led to exile being “reconceptualized as an indefinite transit situation” (ibid., p. 45). She explained how this feeling of uncertainty – or “living in transit” – was affected by conditions in Iraq and Egypt, as well as interactions with governmental and non-governmental organizations; that is, how the micro level is influenced by the meso and macro. For example, she argued that the dual nature of the refugee protection framework, within which the Egyptian government ensures non-refoulement while the UNHCR has assumed many of the responsibilities of the state in terms of assistance management, has contributed to uncertainty (ibid., p. 42).

The study of Brun (2015, p. 31) on internally displaced Georgians from Abkhazia showed how such “protracted uncertainty” can develop to “chronic uncertainty” when time passes more, when “hopes for a possible return in the future have changed from the uncertain to the unforeseeable”.

Several studies, such a Mzayek (2019) and El-Shaarawi (2015, p. 39), dwell on how such situations of waiting and uncertainty impact refugees’ well-being. While such situations might cause suffering, refugees often develop “resilience” or “resilient coping strategies” (Mzayek 2019) during the period of waiting, thus relying on “agency-in-waiting” (Brun 2015).

Some studies link the concept of waiting to refugees’ professional aspirations, contrasting policies/institutional regulations that affect when and what type of work refugees are allowed to do to the refugees’ own experiences or work plans, or as Cangià et al. (2021, p. 57) put it: the “tension between structural constraints defining the temporalities and opportunities of work as a refugee on the one hand, and migrants’ subjective temporal experiences and professional aspirations on the other”. They explained this through the case of highly skilled Syrian refugees and how they react to waiting as a result of the bureaucratic migration/asylum system in Switzerland through their capacity to have “aspirations” (i.e. “ability to construct representations of the *future* through the use of material, relational, emotional or symbolic resources that one can cultivate or has previously accumulated thanks to the opportunities that life has offered in the *past*”) (Cangià et al. 2021, p. 62, emphasis added).

Mozetič (2018, pp. 241, 244) also touched upon the period of waiting and the passivity related to the period following asylum applications and/or the long time (more than two years) it takes to have a medical license of refugee doctors in Sweden (which for non-EU doctors – unlike EU doctors – involved passing several Swedish language courses, then the TULE exam, and then do their residency), and the sense of frustration it causes among them. Relying on Jenkins’s concepts, Mozetič (ibid., pp. 248–249) showed how the labeling or categorization of these people at the institutional level by the legal framework as refugees meant they had to follow strict regulations and this in turn affected the way they self-identified themselves as “foreigners” (or “migrants”) – which they perceived to have less negative connotations – during the initial time while waiting for the approval of their asylum application. She also contrasted refugees’ self-perception as “providers” due to their medical profession with the feeling of being passive “recipient[s]” of social assistance as refugees (ibid., p. 248). A similar point is also made by Cangià et al. (2021, p. 61), where one of the refugees they interviewed had to confront “the construction of an image of the refugee as a migrant who simply benefits from social assistance without being able to contribute or aspire for something better”.

Challenging the “limited” conceptualization of waiting time as “passive, empty and wasted”, especially when linked to institutional or organizational settings, Rotter (2015) explored the experiences of asylum seekers who waited between two and nine years in the United Kingdom for a resolution of their immigration status, and argued that their waiting was “affective, involving a heightened anticipation of the future and reflection on desired and dreaded outcomes; active, as they structured and filled their time with a variety of routines, activities and projects; and, in a more limited sense, productive, as waiting time could be transformed into capital.” Thus, she concluded that, for these asylum seekers, waiting was not just an “empty interlude between events but an intentional and agential process.” Fontanari (2017) also highlighted the interplay between refugees’ and asylum seekers’ agency and the institutional system’s mechanisms. Examining how the European border regime shapes the experience of time in the lives of refugees and asylum seekers, based on ethnographic research in Italy and Germany, she argued that in response to the “intertwining of control and abandonment” in the border system (bureaucratic delays, uncertain asylum decisions, and limited rights stretch out migrants’ periods of waiting), refugees develop “everyday practices of resistance” and strategies to reclaim autonomy over their time, carving out spaces for agency even within “structural constraints”.

Some of the aspects of the kinds of interrelations between the micro and macro levels identified above, from the perspective of temporariness/permanence, are also explained by Griffiths et al. (2013, p. 18) in their review piece:

For example, research on temporary protection mechanisms for refugees demonstrates that immigration policies can restrict people to temporary migration stages, even if these stages become so extended that they would be better characterised as a ‘permanent temporariness’ …, a situation linked to the ‘limbo’ we discuss later. For example, it has been suggested that US immigration policy has been used to keep Salvadoran asylum seekers in an extended temporary condition, via policies such as the granting of ‘Temporary Protected Status’ … Similarly[, ] Anderson has used the concept of ‘precariousness’ to consider the institutional production of insecurity for migrant workers … While in some cases, being temporary may be a source of shame or pain, or even political tool of governance, in other cases, individuals may choose or at least prefer impermanence, reflecting ambiguity towards host societies or a desire to return ‘home’ one day …

The interlinkages between the micro, meso and macro levels are also touched upon by Tschalaer (2023), who argued that waiting within Germany’s asylum system is a sexualized and racialized technology of state power, and that for asylum seekers who are lesbian, gay, bisexual, transgender, queer or questioning, intersex, plus (those with additional identities) (LGBTQI+), this waiting produces distinctive “queer temporalities.” Futures are indefinitely postponed, the present is marked by insecurity and hyper-surveillance, and the past is constantly reactivated through credibility interviews. These queer temporalities disrupt normative life-course assumptions (education, partnership, family formation), producing a sense of temporal disorientation that differs from – yet overlaps with – other asylum seekers’ experiences of waiting. Importantly, Tschalaer (ibid.) showed that waiting is not only oppressive but also contested, as LGBTQI+ asylum seekers actively negotiate, resist and rework temporal constraints imposed by the state; they create queer social spaces and networks; reclaim time through activism, sociality, and mutual care; and strategically navigate asylum timelines and expectations. These practices constitute temporal “resistance,” enabling claimants to carve out liveable presents despite structural uncertainty. From a more meso perspective (dynamics of networks and community supports), this touches upon the topic of resilience, resistance and activism of refugees and asylum claimants as well as the solidarity of citizens expressed throughout their multiple challenges and difficulties, including how different movements attain and retain visibility over time. This topic of refugee activism is, for example, examined by Bhimji (2023). Focusing on 2012–2014 activism at Oranienplatz public square in Berlin, Germany, Bhimji’s article analysed how refugee activities used time, space and visibility to challenge asylum policies, remember resistance, and claim political and social presence in the city. It explored how the meanings attached to the protest and the activists’ strategies changed over time, as the political situation shifted and as public perceptions evolved through changes in media and public awareness.

### (Past, present, ) future

Issues related to past and present have been addressed in different parts above, when discussing the role of memory and nostalgia as well as the lived experiences and perceptions of refugees about their present state, for example. Along these lines and focusing on the micro level, Karageozian (2024) examined the temporal dimensions of past, present and future in the narratives of Armenians in Lebanon – for many of them, decades after their ancestors arrived in Lebanon as refugees; or, for others, following their or their parents’ more recent move to Lebanon from other countries in the region, such as Iraq and Syria, that have experienced wars or other tensions. She argued that “temporality manifests in these narratives mainly through references to past events, the memory of or experiences of actual journeys to ancestral lands, and a sense of uncertainty related to the present and future” (Karageozian 2024, p. 9).

Also at a micro level, the temporal dimension of future has also been alluded above, when examining the concept of uncertainty, for example. El-Shaarawi (2015, p. 50) explained how many Iraqi refugees perceived resettlement as a “promising solution” to their problematic present lives in Egypt. However, they also recognized that it does have its challenges. “While resettlement creates some hope for stability, the resettlement process itself introduces new kinds of uncertainty that could also cause suffering” (ibid.). She also cited the work of Horst (2006) that makes a similar argument, delving on the case of Somali refugees in Kenya. Thus, several studies show how fear, uncertainty, desire, hope and anticipation often coexist in individual refugees’ thoughts and feelings of their and their families’ futures – a seemingly contradictory or strange coexistence of stasis and movement.

The dimension of future from a macro perspective is analysed explicitly in fewer works on refugee studies. This is also the case with social sciences in general and the broad field of migration studies, as Griffiths et al. (2013, pp. 26–27) argued. An important exception in sociology that they highlighted is the work of Barbara Adam on “imagined futures” from a collective perspective and on how modern democracies are elected for short periods of time despite the huge impact of their decisions on the future.^13^ Extending the latter argument to migration studies, Griffiths et al. (2013, p. 27) explained:

That means that governments take decisions that have significant consequences for future generations, but that there is no mechanism for building this in to [sic.] policy decisions, despite calls for migration (and development) policies to take long-term perspectives.

They argued, for example, that migration bureaucracies evaluate “a person’s immigration status at ‘snap shot’ moments in time,” where a migrant is categorized depending on his/her position in the “linear trajectory” of a set “sequence of events from arrival to settlement, productivity, integration and ultimately to naturalisation or return” (ibid., p. 29). I have touched upon this in relation to refugee studies in the “Sequence” sub-section above. But here, I will review the work of Brun, which makes explicit reference to the issue of future from a more macro perspective, and its relations to the micro level.

In one article, Brun (2015) addressed the idea of “hope” and how it is related to “active waiting.” She argued that in the case of protracted internal displacement she examined, the policy category of IDPs “comes to be seen as a social category that restricts people’s opportunities” because “even when people are ‘moving on’ and developing their lives in displacement, they remain fixed within a political status and a humanitarian category that continues to produce uncertain futures” (ibid., p. 20).

In another article, focusing on the case of humanitarian organizations working with Syrian refugees in Jordan, Brun (2016b) explored the role of the future in humanitarianism and how little room there is for it in the current system, with its dominant, taken-for-granted – or “doxic”, in Bourdieusian terms^14^ – logic. Because of the humanitarian system’s emphasis on increased professionalization, the need to create standardized institutions that function based on universalizing ideas and ethical principles, and a common vocabulary, has increased, according to Brun (2016b). However, the downside of such a system is its “inflexibility” in adopting contextualized responses and in adapting dynamically to changing needs and circumstances over time in situations of protracted displacement (Brun 2016b, p. 396). More specifically linked to the dimension of future, Brun (ibid., p. 399) argued that the prevailing humanitarian system places emphasis more on “biological life” than “biographical life”; these approach the concept of future very differently: “Biological life is repetitive, and it consists of activities which arise out of necessity”. This contrasts with biographical life, which is future-oriented, and leads to the development of durable or transformative products. When people are facing imminent conflict, violence, disaster or persecution, it is understandable for the humanitarian system to focus on life-saving decisions and activities (i.e. saving biological life). Nevertheless, Brun (2016b) argued that in situations of protracted humanitarian crises, it is important to look beyond such immediate issues and become more future-oriented. However, the humanitarian system has not developed the proper reasoning and tools to do so, she argued. For example, she explained how humanitarian reason revolves to a large extent around the “emergency imagery” (Brun 2016b, pp. 401–402):

In the emergency imagery, emergencies arise as exceptions to what is understood as otherwise normal social conditions of stability which helps to justify the humanitarian reason. … Thus, humanitarian reason largely stays the same as long as humanitarian work is defined as such: short-term relief work aiming to save strangers’ lives. The reasoning has unintended consequences when crisis become [sic.] the normal, protracted and when there is no return to a stability that may never have been.^15^

Along a similar vein, inspired from the case of Syrian refugees in Lebanon, Fawaz (2017, p. 101) also called for a need to shift from a focus on “humanitarian crisis” or “refugee crisis” to an “urban crisis” with long-standing planning challenges, from a “perception of temporariness and ‘crisis management’” to “the long-term future-oriented approach of planning.”

In addition, Brun (2016b) described how refugee assistance provision often relies on standardized approaches of collecting data (such as the VAF in Jordan, as mentioned in the “Timing” sub-section above) in a snapshot manner, focusing on individual and household needs identified in one specific moment of time fixed in the present. “Through this exercise, refugees are denied a biography and … hope for the future” (ibid., p. 401). Within such a system, “humanitarian workers also in some ways feel stuck,” according to interviews that Brun (ibid., p. 402) conducted. In such circumstances, some of them said they developed their own framework within the general guidelines, relying on their past experiences and knowledge. Brun (ibid., p. 404) gave the example of shelter practices in Jordan, where organizations felt “they had to do something”.

Before concluding this sub-section, it is important to note that it is often hard to make clear distinctions or to study these three dimensions – that is, present, past and future – separately, because they are closely interlinked. Some aspects of these interlinkages are tackled in Omar’s (2022) work. Zetter (1994) also addressed this interplay between the Greek-Cypriot refugees’ present, past and future in relation to the issue of return; the titles of two sub-sections in his article briefly denote these interlinkages: “A Future in the Past: The Wish to Return” and “A Future Without the Past? The Dilemma of Return,” with the wish and the dilemma taking place in the present.

## An initial attempt to advocate for a balanced approach when conceptualizing temporalities in refugee studies: Early exploratory reflections

Having reviewed how different dimensions of time manifest at the micro, meso and macro levels of the social world pertaining to forced displacement, in this concluding section, I call for the need to adopt a balanced approach between mobility and immobility, agency and structure, haphazardness and purposiveness, and informality and formality when studying or trying to understand temporalities in refugee lives and policies. I recognize that these are very complex – and even controversial – concepts in the social sciences. Given their theoretical density, an in-depth elaboration of these concepts is necessary in future elaborations of the initial reflections included in this section. Such an endeavour is not undertaken here, given the paper’s main focus on providing a conceptual review of temporal dimensions in refugee studies (Section Temporal variables for explaining relations between micro, meso and macro levels in refugee studies). Therefore, this section should only be treated as an initial exploratory attempt to briefly point to certain directions for future, more elaborate and detailed conceptualization efforts around these themes.

In certain works addressing temporality issues related to forced displacement, there seems to be an overemphasis on immobility or stasis, or more so on stagnation. Refugees are sometimes represented as being “stuck” in certain situations (life phases, bureaucratic processes, work conditions, physical movement options, etc.), being confined to certain groups or statuses assigned to them by organizations or states through the use of specific labels or categorizations, etc. Linked to this is often a high emphasis placed on the importance and power of seemingly inflexible systems and organizations dealing with refugee issues as immobile structures. In this line of thinking, individual and groups of refugees – as well as individual and groups of policymakers, humanitarian workers, and other practitioners – are represented as quite powerless, lacking the resources, knowledge and capacities to take actions that are meaningful and that might impact the dominant or “doxic” (to use Bourdieu’s term) ideas and practices, that is, to be agents of change. There is, however, another trend, where the agency of individuals and groups, associated with or denoting mobility or movement, is instead highlighted, obscuring to a certain degree the importance of structural dimensions, which might not be always or very easy to maneuver against. Adopting a balanced approach means acknowledging the limits of both extremes, the nuanced nature of these processes, as well as their interrelations. See, for example, how Cangià et al. (2021, p. 57) highlighted and explored “the complex interplay between mobility and immobility,” in line with the (im)mobility perspective.

Advocating for a balanced approach does not mean undermining the significance and power of certain structural or institutional processes or phenomena, or the huge constraints that many refugees face in multiple aspects of their lives. Yet, it also does not mean underestimating the resourcefulness of individuals and groups (whether refugees or policymakers and practitioners) and the evolution of institutions over time. Structures do change over time, and the macro level should not be regarded as a single, unified and stable block acting uniformly. For example, within humanitarian organizations, there are different levels of operations (regional, countrywide, local, etc.); various kinds of activities (advocacy and awareness-raising, physical interventions, capacity-building, evidence generation and knowledge production, etc.); people of varying backgrounds, experiences and expertise and in different positions; diverse sources of funding; etc. Similarly, refugee policymaking and policy implementation by state institutions occur at different levels, with the involvement of different people and the use of various instruments. On the other hand, agency also needs to be understood in a nuanced manner. The latter issue is discussed by Brun (2016a, pp. 2–3). Also, mobility needs to be examined taking into consideration its diverse layers and purposes/motivations, and forms. For example, there are certain forms of temporary, often informal/undocumented, mobility that refugees (especially in situations of protracted displacement) engage in, which could often be disregarded by scholars, humanitarian organizations and states:

This process of informal, often undocumented mobility … pushes at the boundaries and constraints placed on the displaced people by host countries and humanitarian agencies, who perceive it as a denial of a *bona fide* refugee identity. It fits uneasily with notions of quasi-sedentary populations waiting for durable (i.e. permanent) solutions, and a regulated and documented existence within defined boundaries (of the state, of status, of expected behaviour). (Zetter 2011, p. 11, emphasis in original)

The dualities of mobility–immobility and agency–structure are also linked to those of informality and formality, as well as haphazardness and purposiveness (that is, how intentional refugee actions and policies are). As Jenkins (2000, p. 14) argued, “the distinction between informality and formality is never hard and fast”. They co-exist and are highly inter-dependent. Policymaking, for example, includes both formal and informal elements. Thus, the extreme of viewing ambiguity and informality in policy debates/discourse as a sign of complete haphazardness in policymaking should be avoided. For example, Nassar and Stel (2019, p. 44) demonstrated how “institutional ambiguity in the context of the Lebanese response to the Syrian refugee crisis is not merely contingent”, arguing that there also exists “a strategic component to” this ambiguity, linked to a “political utility of maintaining uncertainty and precariousness”. In one of the chapters written by Griffiths (2017) in the book *Timespace and International Migration* edited by Page, Christou and Mavroudi, the author examined the British immigration regulatory system related to “deportable” migrants. She explained how, as the editors summarized in their introductory chapter, “different senses of time (measurable time, linear time, monstrous time, commodified time) co-exist within the bureaucracy not as some analytical accident but for a precise political purpose, which she calls ‘temporal governance’” (Page et al. 2017, p. 9).

At a more macro level, scholarly debates have addressed the extent to which the trajectory of – and the actors in – the so-called international refugee regime, and associated global refugee policy, can be clearly defined, or whether multiple dynamics have shaped it over time. These issues are touched upon in Fakhoury and Chatty’s (2025) recently published edited book on refugee governance in the Arab world, even though the concept of time does not constitute a central/explicit focus of the book. They challenged the image of Arab states’ legal and asylum systems, mostly predominant in policy and news analyses, as being “in *counter position* to rather than in *juxtaposition* with the evolution of the modern refugee regime” (ibid., p. 7). Instead, they put forward an alternative narrative that acknowledges the “multiplicity of refugee lawmaking and agenda-setting histories” beyond the often Western-driven refugee order (ibid., p. 5). They did so by conceptualizing the engagement of Arab states with the international refugee regime “as multi-centric, variegated and diverse, relying on a variety of top-down, side-by-side, network, bottom-up and everyday governance structures” (ibid., p. 12), while also exploring how refugees themselves are to navigate such a complex governance structure.

Recent attempts have been made to empirically study the relationship between institutional strategies and the lived experiences of refugees. Shapiro et al. (2025), for example, highlighted the intersection between “temporal governance” leading to “acceleration, entrapment and rupture” and the “temporal agency” refugees exert while engaging with the consequences of these different dimensions of governance, using different “tactics,” looking as well at the relatively lesser-studied meso level (e.g. refugee families, intergenerational issues).

But how can we build on such studies, while adopting a balanced approach between mobility and immobility, agency and structure, haphazardness and purposiveness, and informality and formality when conceptualizing temporalities in refugee studies?

From a more micro perspective, a more nuanced understanding of the concept of human agency might be helpful, as briefly mentioned above. Such an attempt has been made recently by Tefera and Gamlen (2025, pp. 982–983), who introduced the concept of “experiential time … to capture the subjective, fluid, and multifaceted temporalities that refugees navigate”. They identified four categories of experiential time – *“sluggish*,* stuck*,* racing*, and *shock*,” each reflecting “the ways in which speed and duration interact to produce unique emotional and psychological responses” (ibid., pp. 982–983, emphasis in original). It might be useful to extend such attempts by taking into consideration other dimensions of time discussed above in this paper. The recent introduction of the concept of “temporal logics” by Tefera and Gamlen (2024) advances in that direction, drawing partly on Rowell et al. (2016)’s work on “temporal structures,” which identified three dimensions of time – “concepts”, “orientations” and “patterns”. While Rowell et al. (2016)’s work emphasized structure over agency, according to Tefera and Gamlen (2024, p. 864), their proposed concept of temporal logics, they argued, presents a more comprehensive framework that also takes into account “not only collective and sociological elements of temporal organization but also affective and cognitive constructs and responses related to the perception, passage, and use of time”. The concept of temporal logics, according to Tefera and Gamlen (2024, p. 862), helps examine “the intricate ways migrants’ experiences intertwine with temporal factors, such as duration of displacement, timing of events, frequency of movement, periods of waiting, seasonal cycles, and the pace of life changes”.

It might be helpful to build upon such conceptual frameworks by exploring links with or insights from other broader social theory works that present nuanced understandings of the concept of human agency. One such example is the work of Emirbayer and Mische (1998). They conceptualized agency as encompassing the “*interplay of habit*,* imagination*,* and judgment*” that “*both reproduces and transforms*” structural environments within which actors are engaged temporally and relationally. Through this definition, they took into consideration three dimensions and corresponding temporal orientations of agency that co-exist in diverse degrees: “iteration” or routine (past), “projectivity” or purposivity (future), and “practical evaluation” or judgment (present) (emphasis in original) (ibid., p. 970).^16^ From a more macro perspective, another source of potential insights could be studies that explain change in policymaking (See Howlett and Goetz 2014). The literature on “intentional and unintentional sequencing” could be particularly helpful in this regard. See, for example, also Howlett (2019)’s article. Moreover, a greater focus on the meso level might enrich these conceptual works.

Finally, the above-described balanced approach to the conceptualization of time in refugee studies should also be extended to the relationship between time and space. Forced displacement studies have often dealt with “place as mere context” (Fawaz et al. 2022) or as highly immobile or static, ignoring the dynamic nature of space and of people’s perceptions of space over time. More attention should be placed on a balanced approach when studying time–space relations, taking into consideration the mobilities and immobilities of spaces. “An integrated reading of place” that recognizes “spatial transformations,” along with static dimensions of space, is highly relevant especially when studying forced displacement in urban settings, where refugee and host populations interact with each other and the boundaries between them as distinct groups are often blurred (ibid.). From a policy perspective, “area-based approaches” to humanitarian, recovery and development programming or action in areas affected by urban displacement (Schell et al. 2020) might present a useful lens in this regard, especially if they successfully account for temporal perspectives and if they recognize the complex co-existence and interplay of the different levels and aspects of agency by individuals and social groups with structural dimensions.

## Conclusion

This paper argued that recent scholarship has advanced the analysis of temporality across migration studies; however, a focused conceptual synthesis for refugee studies remains scarce. Reviewing temporality systematically offers important analytical leverage for refugee studies, a subfield in which time has often been present but insufficiently theorized. By reviewing how core temporal variables – timing; sequence (or order); frequency (or rhythm), including (a)synchronization; speed (or tempo); duration; and past, present and future – have been taken up across selected strands of scholarship, the paper has demonstrated how refugee lives and policies are shaped through interlocking temporal processes operating at micro, meso and macro levels. Taken together, the reviewed works show that temporality is not merely an experiential backdrop to forced displacement but a constitutive element of power, governance, identity formation, and social relations. At the same time, the analysis underscores the need to resist one-sided or reductive temporal framings. Building on the reviewed literature and recognizing the empirical entanglement of temporal variables and levels of analysis, this paper has offered an initial attempt to advocate for a more balanced approach to conceptualizing temporalities in refugee studies – one that avoids privileging one or the other of such complex binaries as immobility and mobility, structure and agency, formality and informality, and purposiveness and haphazardness. Future research could extend these reflections by exploring relatively underexamined temporal dimensions or levels of the social world (e.g. meso), unpacking further the above-mentioned binary concepts, more fully examining implications of temporal perspectives for policy design and humanitarian practice, etc. Doing so would not only deepen conceptual debates but also contribute to more context-sensitive and future-oriented understandings of refugee lives and protection regimes.

## Data Availability

No datasets were generated or analysed as part of this paper.

## Footnotes

1 He focuses on the concept of “time geography”.

2 See Cwerner (2001) for a key article, where after providing a review of previous relevant studies, he proposes a conceptual framework based on the development of the concepts of the “strange, heteronomous, asynchronous, remembered, collage, liminal, diasporic and nomadic times of migration”.

3 Here, I acknowledge long-standing debates in legal, policy, academic, etc. circles concerning the different labels/categorization used in the topic/study of migration – such as “migrants”/“refugees”, “economic migration”/”forced migration” – as well as the politics of naming, including efforts or arguments that problematize and deconstruct rigid or clear-cut distinctions between these labels or categories (e.g. Malkki 1995; Zetter 1991, 2007). While this paper recognizes that such labels cannot fully capture all the complexity and nuances of lived experiences, it also acknowledges that certain distinctions remain analytically meaningful and legally consequential, and therefore cannot be entirely disregarded, especially for conceptual, legal, policy or advocacy purposes.

4 Adam (2008, pp. 7–8) defined these elements briefly as follows: • “*Time frame* – bounded, beginning and end of day, year, life time, generation, historical/geological epoch; • *Temporality* – process world, internal to system, ageing, growing, irreversibility, directionality; • *Timing* – synchronisation, co-ordination, right/wrong time; • *Tempo* – speed, pace, rate of change, velocity, intensity: how much activity in given timeframe; • *Duration* – extent, temporal distance, horizon: no duration = instantaneity, time point/moment; • *Sequence* – order, succession, priority: no sequence = simultaneity, at same time; • *Temporal modalities*: past, present and future – memory, perception/experience and anticipation.”

5 In such cases, I have not cited or made explicit reference to all relevant works that I have read while preparing this paper, both to avoid excessive repetition of ideas and overly imbalanced treatment of the different themes.

6 Similarly, a study exists in Lebanon, the Vulnerability Assessment of Syrian Refugees (VASyR), which is conducted annually by the United Nations High Commissioner for Refugees (UNHCR), the United Nations Children’s Fund (UNICEF), and the United Nations World Food Programme (WFP).

7 Omar’s work is also relevant to the sub-section of “Present, Past, Future”.

8 These themes are also linked to the dimensions of “past, present and future”, as well as the topic of “waiting” (examined mainly under “speed” and “duration”).

9 For a summary of these concepts, and how they apply to migration studies, see Karageozian (2015).

10 Griffiths et al. (2013, p. 15) referred to some aspects of duration in their section on “flows”.

11 Besides the studies mentioned in this section, see also other works on waiting related to displacement, listed by Brun (2015, p. 19).

12 In this regard, Mzayek (2019) cited the works of Malkki (1995) and Turton (2004).

13 See Griffiths et al. (2013, pp. 26–27), for references of Adam’s work in this regard.

14 As mentioned above, for a summary of Bourdieusian social theory concepts, including that of “doxa,” and how they apply to migration studies, see Karageozian (2015).

15 Referring to protracted displacement of both IDPs and refugees, Zetter (2011) also referred to some of these issues.

16 For more information about their definition, see a summary, and how it can be applied to migration studies, in Karageozian (2015).

## Abbreviations

IDPs: Internally displaced persons
LGBTQI+: Lesbian, gay, bisexual, transgender, queer or questioning, intersex, plus [those with additional identities]
TPD: Temporary Protection Directive
UK: United Kingdom
UNHCR: United Nations High Commissioner for Refugees
UNICEF: United Nations Children’s Fund
USA: United States of America
U.S.: United States
VAF: Vulnerability Assessment Framework
VASyR: Vulnerability Assessment of Syrian Refugees [in Lebanon]
WFP: [United Nations] World Food Programme

## References

[CR1] Adam B (1998) Timescapes of modernity. Routledge, London

[CR2] Adam B (2004) Time. Polity, Cambridge, UK

[CR3] Adam B (2008) The timescapes challenge: Engagement with the invisible temporal, pp. 7–12. In: Adam B, Hocke, J, Thompson P, and Edwards R (ed) Researching lives through time: Time, generation and life stories. Timescapes Working Paper Series, June, No. 1, University of Leeds

[CR4] Andersson R (2014) Time and the migrant other: European border controls and the temporal economics of illegality. Research Papers in Economics

[CR5] Beirens H, Hughes N, Hek R, Spicer N (2007) Preventing social exclusion of refugee and asylum seeking children: Building new networks. Social Policy and Society 6(2):219–22

[CR6] Benezer G, Zetter R (2014) Searching for directions: Conceptual and methodological challenges in researching refugee journeys. Journal of Refugee Studies 28(3):297–318

[CR7] Bhimji F (2023) The temporalities of refugee activism: Mutating meanings and visibility while remembering resistance and claiming place at Oranienplatz. Journal of Immigrant and Refugee Studies 23(2):225–237

[CR8] Brun C (2015) Active waiting and changing hopes: Toward a time perspective on protracted displacement. Social Analysis 59(1), Spring :19–37

[CR9] Brun C (2016a) Dwelling in the temporary: The involuntary mobility of displaced Georgians in rented accommodation. Cultural Studies 30(3): (Im)Mobilities of Dwelling: Places and Practices: 421–440

[CR10] Brun C (2016b) There is no future in humanitarianism: Emergency, temporality and protracted displacement. History and Anthropology 27(4):393–410

[CR11] Cangià F, Davoine E, Tashtish S (2021) (Im)mobilities, waiting and professional aspirations: The career lives of highly skilled Syrian refugees in Switzerland. Geoforum 125:57–65

[CR12] Corcodel V, Cardoso L, Felgueiras M (eds) (2023) The Temporary Protection Directive in the context of the 2015/2016 and the 2022/2023 ‘crises’: Geopolitics, race or both? CEDIS. https://novarefugeelegalclinic.novalaw.unl.pt/?blog_post=the-temporary-protection-directive-in-the-context-of-the-2015-2016-and-the-2022-2023-crises-geopolitics-race-or-both

[CR13] Cwerner SB (2001) The times of migration. Journal of Ethnic and Migration Studies 27(1):7–36

[CR14] El-Shaarawi N (2015) Living an uncertain future temporality, uncertainty, and well-being among Iraqi refugees in Egypt. Social Analysis: The International Journal of Anthropology 59(1), Spring: 38–56

[CR15] Emirbayer M, Mische A (1998) What is agency? The American Journal of Sociology 103(4), January: 962–1023

[CR16] Fakhoury T, Chatty D (eds) (2025) Refugee governance in the Arab World: The international refugee regime and global politics. I.B. TAURIS, London and New York

[CR17] Fantino AM, Colak A (2001) Refugee children in Canada: Searching for identity. Child Welfare LXXX(5), September: 587–596 11678416

[CR18] Fawaz M (2017) Planning and the refugee crisis: Informality as a framework of analysis and reflection. Planning Theory 16(1):99–115

[CR19] Fawaz M, Al-Hage C, Harb M (2022) Unplanned links, unanticipated outcomes: Urban refugees in Halba (Lebanon). Society and Space 40(3):486–507

[CR20] Fontanari E (2017) ‘It’s my life’. The temporalities of refugees and asylum-seekers within the European border regime. Etnografia e ricerca qualitativa (ERQ) 1:25–54

[CR21] Fozdar F, Hartley L (2014) Civic and ethno belonging among recent refugees to Australia. Journal of Refugee Studies 27(1), March: 126–144

[CR22] Grabska K (2020) ‘Wasting time’: Migratory trajectories of adolescence among Eritrean refugee girls in Khartoum. Critical African Studies 12(1):22–36

[CR23] Griffiths M (2017) The changing politics of time in the UK’s immigration system. In: Mavroudi E, Page B, Christou A (eds) Timespace and international migration. Edward Elgar Publishing Limited, Cheltenham and Northampton, pp 48–60

[CR24] Griffiths M, Rogers A, Anderson B (2013) Migration, time and temporalities: Review and prospect. COMPAS Research Resources Paper, March

[CR25] Guillemette L, Lévesque C (n.d.), Narratology, Signo. http://www.signosemio.com/genette/narratology.asp

[CR26] Haas BM (2017) Citizens-in‐waiting, deportees‐in‐waiting: Power, temporality, and suffering in the US asylum system. Ethos 45(1):75–97

[CR27] Heidbrink L (2022) ‘How can I have a future?’: The temporal violence of deportation. Journal of Intercultural Studies 43(4):480–496

[CR28] Horst C (2006) *Buufis* amongst Somalis in Dadaab: The transnational and historical logics behind resettlement dreams. Journal of Refugee Studies 19(2):143–157

[CR29] Horst C, Grabska K (2015) Introduction: Flight and exile—Uncertainty in the context of conflict-induced displacement. Social Analysis: The International Journal of Anthropology 59(1), Spring: 1–18

[CR30] Hovil L (2016) Refugees, conflict and the search for belonging. Palgrave Macmillan, Cham

[CR31] Howlett M (2019) Procedural policy tools and the temporal dimensions of policy design: Resilience, robustness and the sequencing of policy mixes. International Review of Public Policy 1(1):27–45

[CR32] Howlett M, Goetz KH (2014) Introduction: Time, temporality and timescapes in administration and policy. International Review of Administrative Sciences 80(3):477–492

[CR33] Ineli-Ciger M (2022) Reasons for the activation of the Temporary Protection Directive in 2022: A tale of double standards. Forum on the EU Temporary Protection Responses to the Ukraine War. https://www.asileproject.eu/reasons-for-the-activation-of-the-temporary-protection-directive-in-2022-a-tale-of-double-standards/

[CR34] Jenkins R (2000) Categorization: Identity, social process and epistemology. Current Sociology 48(3):7–25

[CR35] Karageozian N (2015) Long-term diasporic return migration in Post-Soviet Armenia: Balancing mobility and sedentarism (doctoral thesis), Oxford: University of Oxford. https://ora.ox.ac.uk/objects/uuid:25ff00d2-816b-4fdd-b8fb-ec5eeb4ceead/files/md45dcf179d3ed826529f7d7e1eee3920

[CR36] Karageozian N (2024) Temporalities in diasporic voices: Past, present and future in narratives of Armenians in Lebanon. Rights for Time, Policy and Time Strand (Lebanon): Working Paper. https://rights4time.com/project/policy-and-time/#flipbook-df_1021/1/

[CR37] Khosravi S (2020) Afterword: Waiting, a state of consciousness. In: Jacobsen CM, Karlsen M-A, Khosravi S (eds) Waiting and the temporalities of irregular migration, 1st edn. Routledge, London and New York, pp 202–208

[CR38] King R, Thomson M, Fielding T, Warnes T (2004) Gender, age and generations: State of the art report Cluster C8. SCMR-Sussex Centre for Migration and Population Studies. University of Sussex, December

[CR39] Kobelinsky C (2021) Life while waiting: Experiencing the asylum application in France. In: Mercier D, Zuñiga V, Doraï K, El Miri M, Peraldi M (eds) Experiencing ruptures in migration: The ordinary and unexpected journeys of global migrants. Transnational Press London, London, pp 117–130

[CR40] Malkki LH (1995) Purity and exile: Violence, memory, and national cosmology among Hutu refugees in Tanzania. University of Chicago Press, Chicago, Illinois

[CR41] Martin T (2016) Temporality and literary theory. Oxford Research Encyclopedia of Literature. Oxford University Press USA, p 2

[CR42] Mozetič K (2018) Being highly skilled and a refugee: Self-perceptions of non-European physicians in Sweden. Refugee Survey Quarterly 37:231–251

[CR43] Mzayek M (2019) Understanding waiting and wellbeing through liminal experiences of Syrian refugees. Migration Letters 16(3):369–377

[CR44] Näre L, Tkach O (2024) The temporary protection of Ukrainian refugees: A model for the future or a case of discriminatory exceptionalism? Mixed Migration Centre. https://mixedmigration.org/temporary-protection-ukrainian-refugees/

[CR45] Nassar J, Stel N (2019) Lebanon’s response to the Syrian refugee crisis – Institutional ambiguity as a governance strategy. Political Geography 70:44–54

[CR46] Novak P (2017) Border rhythms. In: Mavroudi E, Page B, Christou A (eds) Timespace and international migration. Edward Elgar Publishing Limited, Cheltenham and Northampton, pp 61–76

[CR47] Omar L (2022) Foreclosed futures and entangled timelines: Conceptualization of the ‘future’ among Syrian newcomer mothers in Canada. Journal of Ethnic and Migration Studies 49(5):1210–1228

[CR48] Page B, Christou A, Mavroudi E (2017) Introduction: From time to timespace and forward to time again in migration studies. In: Mavroudi E, Page B, Christou A (eds) Timespace and international migration. Edward Elgar Publishing Limited, Cheltenham and Northampton, pp 1–16

[CR49] Rotter R (2015) Waiting in the asylum determination process: Just an empty interlude? Time & Society 25(1):80–101

[CR50] Rowell C, Gustafsson R, Clemente M (2016) How institutions matter “in time”: The temporal structures of practices and their effects on practice reproduction. How Institutions Matter! Research in the Sociology of Organizations 48A, Emerald Group Publishing Limited, Leeds: pp 303–327

[CR51] Schell J, Hilmi M, Hirano S (2020) Area-based approaches: An alternative in contexts of urban displacement. Forced Migration Review 63, February

[CR53] Schon J (2019) Motivation and opportunity for conflict-induced migration. Journal of Peace Research 56(1):12–27 Special Issue on Refugees, Forced Migration, and Conflict (January)

[CR52] Shapiro D, Syppli Kohl K, Jørgensen RE, Sandberg M (2025) Out of sync: Temporal governance and agency in refugee families with temporary protection status in Denmark. Journal of Refugee Studies 38(1):165–180

[CR54] Tarkhanova O, Pyrogova D (2024) Forced displacement in Ukraine: Understanding the decision-making process. European Societies 26(2):481–500

[CR55] Tefera GW, Gamlen A (2023) Temporal logics in urban place-making: The case of refugee-background Ethiopians in Australia. Urban Geography 44:243–261

[CR56] Tefera GW, Gamlen A (2024) Temporal logics in geographical research on migration and refugees. Progress in Human Geography 48(6):861–878

[CR57] Tefera GW, Gamlen A (2025) Theorizing experiential time in refugee migration: Speed and duration. Journal of Refugee Studies 38(4):982 –997

[CR58] Tschalaer M (2023) Queering migration temporalities: LGBTQI+ experiences with waiting within Germany’s asylum system. Ethnic and Racial Studies 46(9):1833–1853

[CR59] Turton D (2004) The m eaning of place in a world of movement: Lessons from long-term field research in Southern Ethiopia. Working Paper no. 18, Oxford: Refugee Studies Centre, University of Oxford

[CR60] van Blerk L, Shand W, Prazeres L, Bukenya B, Essaid AA, Hunter J, Ibrahim RW, Kasirye R (2021) Youth transitions in protracted crises: Conceptualising the ‘rupture’ of refugees’ pathways to adulthood in Uganda and Jordan. Transactions of the Institute of British Geographers (Trans Inst Br Geogr) 00:1–16

[CR63] Zetter R (1991) Labelling refugees: Forming and transforming a bureaucratic identity. Journal of Refugee Studies 4(1):39–62

[CR62] Zetter R (1994) The Greek-Cypriot refugees: Perceptions of return under conditions of protracted exile. International Migration Review 28(2), Summer:307–322

[CR64] Zetter R (2007) More labels, fewer refugees: Remaking the refugee label in an era of globalization. Journal of Refugee Studies 20(2):172–192

[CR65] Zetter R (2011) Unlocking the protracted displacement of refugees and internally displaced persons: An overview. Refugee Survey Quarterly 30(4):1–13

